# The Emerging Role of RNA N6-Methyladenosine Modification in Pancreatic Cancer

**DOI:** 10.3389/fonc.2022.927640

**Published:** 2022-07-22

**Authors:** Xiaoge Hu, Xiangxiang Lei, Jinhui Guo, Wen Fu, Wen Sun, Qiliang Lu, Wei Su, Qiuran Xu, Kangsheng Tu

**Affiliations:** ^1^ Department of Hepatobiliary Surgery, The First Affiliated Hospital of Xi’an Jiaotong University, Xi’an, China; ^2^ The Key Laboratory of Tumor Molecular Diagnosis and Individualized Medicine of Zhejiang Province, Zhejiang Provincial People’s Hospital, Affiliated People’s Hospital, Hangzhou Medical College, Hangzhou, China; ^3^ Department of Hepatobiliary and Pancreatic Surgery, Zhejiang Provincial People’s Hospital, Affiliated People’s Hospital, Hangzhou Medical College, Hangzhou, China; ^4^ Institute of Basic Medicine and Forensic Medicine, Hangzhou Medical College, Hangzhou, China; ^5^ Qingdao Medical College, Qingdao University, Qingdao, China; ^6^ The Second Clinical Medical College, Zhejiang Chinese Medical University, Hangzhou, China; ^7^ Department of Hepatobiliary and Pancreatic Surgery, The First Affiliated Hospital, Zhejiang University School of Medicine; Zhejiang Provincial Key Laboratory of Pancreatic Disease; Innovation Center for the Study of Pancreatic Diseases, Hangzhou, China

**Keywords:** pancreatic cancer, non-coding RNAs, coding RNAs, RNA methylation, N6-methyladenosine (m6A)

## Abstract

Pancreatic cancer (PC) is one of the most common malignant cancers, ranking the seventh highest causes of cancer-related deaths globally. Recently, RNA N6-methyladenosine (m6A) is emerging as one of the most abundant RNA modifications in eukaryote cells, involved in multiple RNA processes including RNA translocation, alternative splicing, maturation, stability, and degradation. As reported, m6A was dynamically and reversibly regulated by its “writers”, “erasers”, and “readers”, Increasing evidence has revealed the vital role of m6A modification in the development of multiple types of cancers including PC. Currently, aberrant m6A modification level has been found in both PC tissues and cell lines. Moreover, abnormal expressions of m6A regulators and m6A-modified genes have been reported to contribute to the malignant development of PC. Here in this review, we will focus on the function and molecular mechanism of m6A-modulated RNAs including coding RNAs as well as non-coding RNAs. Then the m6A regulators will be summarized to reveal their potential applications in the clinical diagnosis, prognosis, and therapeutics of PC.

## Introduction

Pancreatic cancer (PC) is the seventh highest leading cause of cancer mortality worldwide accompanied by poor prognosis as well as a 5-year survival rate of about 10% (1, 2). With the development of clinical diagnosis and treatment for PC in the past two decades, the survival rate of PC patients has increased yearly, while the mortality rate of PC patients remains high. According to the statistics, the death cases (466,000) of PC are close to its new cases (496,000) globally (1). Lacking typical clinical symptoms and early diagnosis biomarkers, numerous PC patients are diagnosed at an advanced stage and miss the chance to get a surgical resection, resulting in the worse clinical outcome of PC patients. Thus, there is great urgency to clarify the initiation and progression of PC. Currently, aberrant genetic mutations (KRAS, p53, CDKN2A, SMAD4), dysregulation of the key signaling pathway (TGF-β, Wnt/β-catenin, Notch, Hippo, YAP), and epigenetic alterations (DNA methylation, RNA methylation, posttranslational modifications) have been reported to participate in PC development (3, 4). However, the molecular mechanism of PC progression remains largely unknown. Therefore, a comprehensive understanding of the pathogenesis and molecular regulatory mechanism of PC will greatly contribute to the early diagnosis, prognosis, and targeted therapeutics development of PC.

In recent years, RNA modifications, such as N6-methyladenosine (m6A), 5-methylcytosine (m5C), N1-methylguanosine(m1A), have been extensively reported in many cancers including PC (5). As one of the most abundant RNA modifications in eukaryotes, m6A has attracted more and more attention, which existed in RNAs including protein-coding RNAs as well as non-coding RNAs (ncRNAs). As reported, m6A modification is catalyzed by the methyltransferase (also called “m6A writers”) and meanwhile can also be removed by the demethylases (also called “m6A eraser”) (5, 6). Additionally, m6A-binding proteins (also called “m6A readers”) recognize and bind to the m6A-modified RNAs to regulate RNA fate (5). In PC, a significantly increased m6A level has been observed in both PC tissues and cell lines, and an elevated m6A level was associated with poor prognosis of PC patients (7–11). So far, dysregulation of m6A-associated modulators and m6A-modified RNAs has been associated with PC cell growth, iron metabolism, glycolysis metabolism, stemness-like property, and metastasis.

In this review, we will systemically summarize the molecular mechanisms and biological functions of m6A modifications in both mRNA and ncRNAs as well as the m6A regulators in the initiation and progression of PC and then discuss the potential applications of m6A modifications in the clinical diagnosis, prognosis, and targeted therapy of PC.

## m6A Modification

**Figure 1:**
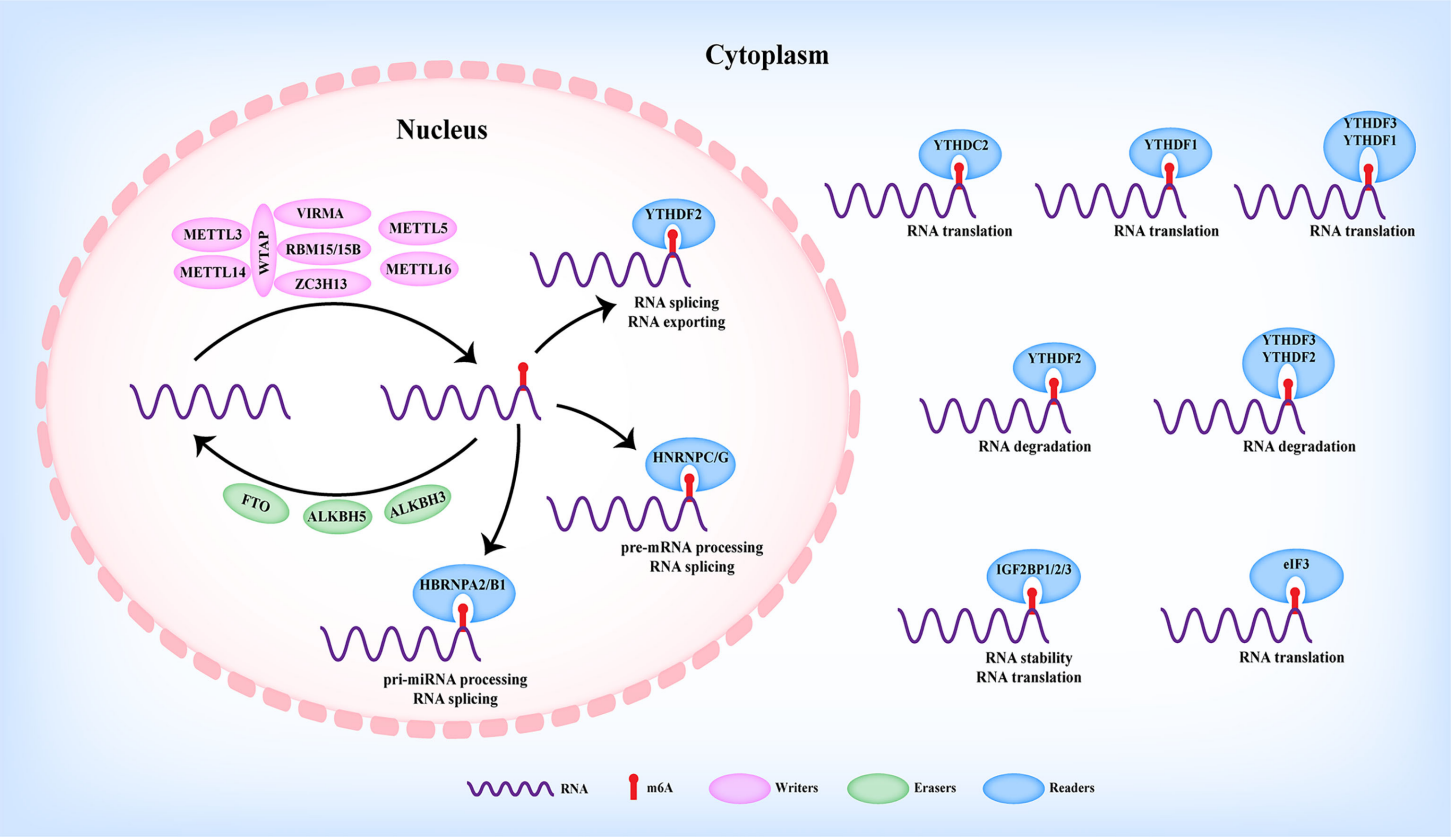
The dynamic regulation of m6A modification in RNAs.

The RNA N6-methyladenosine (m6A) modification was defined to methylate the N6 position of adenosine, which was firstly reported in eukaryotic cells by Desrosiers et al. in 1974 (12). Up to now, m6A modification was considered to be the most abundant modification in RNAs (13, 14) and prefers to occur at the consensus motif RRACH (R=A or G, H=A, C, or U) of 3′-untranslated regions (3′UTRs), long internal exons (CDS), and near stop codons rather than randomly happens (15, 16). Nowadays, with the development of m6A detection-associated technologies, m6A modifications have been revealed in various types of RNAs including protein-coding RNA and ncRNAs, such as long non-coding RNAs (LncRNAs), microRNAs (miRNAs), circular RNAs (circRNAs), ribosomal RNA (rRNA), and transfer RNA (tRNA) (5, 17, 18). As shown in **Figure 1**, m6A modifications have been shown to be involved in RNA processes including nuclear export, miRNA maturation, alternative splicing, stability, translation, and degradation, thus participating in the initiation and progression of various diseases (19). As reported, m6A modification was dynamically and reversibly regulated by m6A methyltransferase (“writers”), m6A demethylases (“erasers”), and m6A-binding proteins (“readers”) (**Figure 1**) (20).

### Writers

m6A modification was installed by the methyltransferase complex (MTC), in which Wilms’ tumor-1-associated protein (WTAP), methyltransferase-like 3 (METTL3), and methyltransferase-like 14 (METTL14) formed the core component (**Figure 1**). METTL3 was the firstly identified m6A writer with catalytic activity to trigger m6A modification *via* the S-adenosylmethionine (SAM)-binding motif. Serving as a supporting structure without catalytic activity, METTL14 formed a METTL3/METTL14 complex with METTL3 to recognize the conserved RRACH motif (21, 22). WTAP was further revealed to interact with the METTL3/METTL14 complex to mediate their nuclear speckle localization, thus modulating target RNA m6A modification (23). Other m6A readers including Vir-like m6A RNA methyltransferase-associated protein (VIRMA/KIAA1429), RNA-binding motif protein 15/15B (RBM15/15B), and zinc finger CCCH domain-containing protein 13 (ZC3H13) have also been identified to participate in the m6A modification of MTC (**Figure 1**). For example, VIRMA recruited the catalytic METTL3/METTL14/WTAP complex to mediate m6A modification in the 3′UTR and near the stop codon (24). RBM15/15B could interact with and recruit the MTC to a specific site to enhance the m6A modification of the LncRNA X-inactive specific transcript (XIST), thereby facilitating XIST-mediated gene silencing (25). Moreover, ZC3H13 enhanced the nuclear translocation of the ZC3H13–WTAP–Virilizer–Hakai complex to facilitate m6A modification (26). Apart from the above writers, methyltransferase-like protein 5 (METTL5) and methyltransferase-like protein 16 (METTL16) were identified as m6A writers (**Figure 1**) (27–30).

### Erasers

Contrary to the function of writers, m6A erasers exerted the demethylation of m6A modification of RNAs. Currently, erasers mainly contain fat mass and obesity-associated protein (FTO), alkB homolog 5 (ALKBH5) (**Figure 1**) (31, 32), both of which are primarily located in the nucleus and belong to the alkB family (32). As reported, FTO was the first identified eraser, participating in the m6A modification of nuclear RNAs (31). To date, FTO and ALKBH5 have been widely reported in modulating RNA m6A modification in various human cancers (19). Recently, alkB homolog 3 (ALKBH3) was revealed as a novel m6A eraser in mediating tRNA demethylation and protein translation (**Figure 1**) (33).

### Readers

Unlike m6A writers or m6A erasers to add or remove the m6A modification of RNAs, readers could recognize and interact with m6A-motified RNAs, thereby modulating various RNA processes, such as alternative splicing, nuclear export, miRNA maturation, stability, degradation, and translation (**Figure 1**) (34). Currently, insulin-like growth factor 2-binding proteins (IGF2BPs), YTH family proteins (YTHDC1/2 and YTHDF1/2/3), heterogeneous nuclear ribonucleoprotein family (HNRNPC, HNRNPG, HNRNPA2B1), and eIF3 have been identified as m6A readers (**Figure 1**). Based on their cellular localization, m6A readers exerted different functions. On the one hand, m6A readers with nuclear localization including YTHDC1 (35–38), HNRNPA2B1 (39, 40), and HNRNPC/G (41, 42) were reported to be involved in pri-miRNA processing, splicing, and nuclear exporting of m6A-modified RNAs (**Figure 1**). On the other hand, m6A readers with cytoplasmic localization, such as YTHDF1 (43), YTHDF2 (44, 45), YTHDF3 (46, 47), YTHDC2 (48, 49), IGF2BPs (50), and eIF3 (51), were demonstrated to participate in the stability, translation, and degradation of m6A-modified RNAs (**Figure 1**). In addition, CCHC-type zinc finger nucleic-acid binding protein (CNBP) (52) and NF-κB associated protein (NKAP) (53) were recently uncovered as novel m6A readers involved in modulating stability and pri-miRNA processing.

## Role of RNA m6A Modification in PC

### m6A Modification of Coding RNAs in PC

#### WIF-1

Wnt inhibitory factor 1 (WIF-1) was firstly identified by Hsieh et al. as a secreted Wnt-binding protein to suppress Wnt signaling activity (54). Currently, the tumor-suppressive role of WIF-1 has been clarified in various cancers including PC (55–58).

**Figure 2:**
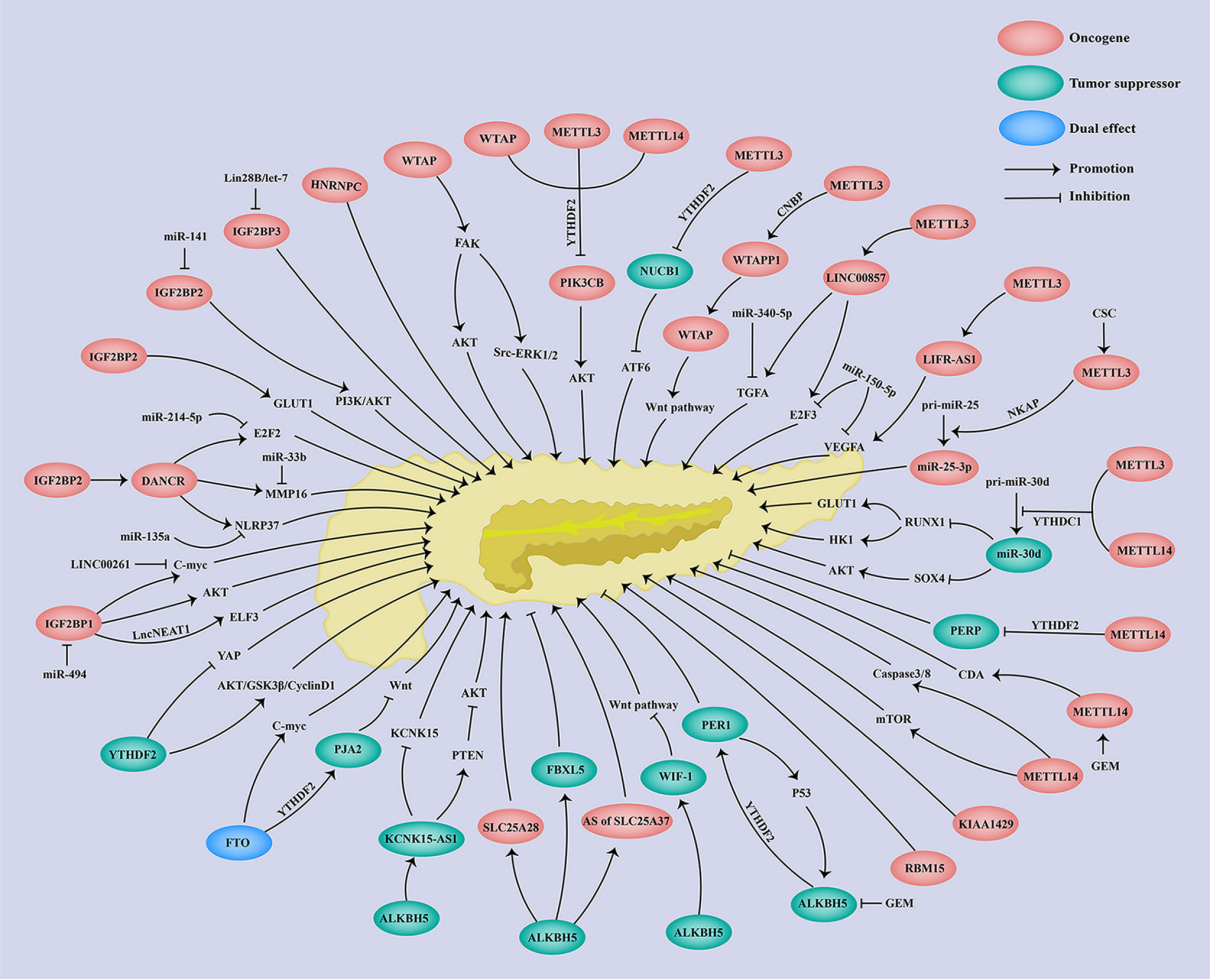
Regulation network of m6A regulators and associated genes in pancreatic cancer.

As shown in **Table 1**, the WIF-1 protein level was downregulated in PC tissues, which was correlated with poor overall survival (OS) of PC patients (59). WIF-1 was further identified as a downstream target of ALKBH5 *via* m6A-seq and RNA-seq. The demethylase-ALKBH5 increases WIF-1 expression through reducing the m6A level of WIF-1 mRNA, which inhibits the Wnt signaling pathway *via* the WIF-1/Wnt axis. Functionally, knockdown of WIF-1 alleviated the ALKBH5-induced suppression of cell growth, migration, and invasion, while overexpression of WIF-1 attenuated the ALKBH5 deficiency-induced promotion of cell growth, migration, and invasion (56) (**Table 1**; **Figure 2**). All the above findings suggests the antitumor role of WIF-1 in the malignant progression of PC.

**Table 1:** m6A modification of mRNAs in pancreatic cancer.

Name	Role	Expression	Function	Clinical significance	m6A regulator	Mechanisms	Refs
WIF-1	Suppressor	Down	WIF1 inhibited cell proliferation, migration, and invasion	OS↑	ALKBH5	1.ALKBH5 decreased the m6A level of WIF-1 and increasesdWIF-1 expression, thus suppressing the Wnt signaling pathway *via* the AKLBH5/WIF-1/Wnt axis.	(56, 59)
PER1	Suppressor	Down	PER1 Inhibited cell proliferation and invasion	OS↑	ALKBH5 YTHDF2	1.ALKBH5 decreased the m6A level of PER1 mRNA and increased the PER1 expression. 2.YTHDF2 mediated PER1 mRNA degradation in an m6A-dependent way.	(11)
	Oncogene	Up	PER1 KD inhibited PC cell growth	–	–	1.TNF-α decreased PER1 expression	(60, 61)
PERP	Suppressor	Down	PERP Inhibited cell proliferation, migration, and invasion	–	METTL14 YTHDF2	1.METTL14 KD decreased the m6A level of PERP 3′UTR and increased the PERP expression 2.YTHDF2 recognized the m6A of PERP 3′UTR to promote PERP mRNA degradation.	(7, 62)
PIK3CB	Oncogene	Up	PIK3CB promoted the cell proliferation, migration, invasion, metastasis, and tumorigenesis of PTEN-deficient PC cells.	OS↓ DFS↓	METTL3 METTL14 WTAP YTHDF2	1.Knockdown of METTL3/METTL14/WTAP separately reduced the m6A level of PIK3CB while it increased PIK3CB expression. 2. YTHDF2 recognized and bound to m6A-modified PIK3CB to induce PIK3CB mRNA decay. 3.PIK3CB activated the Akt signaling pathway.	(63, 64)
PJA2	Suppressor	Down	PJA2 KD promoted PC cell growth, migration, and invasion *in vitro*	–	FTO YTHDF2	1.FTO reduced the m6A level of PJA2 and increased the PJA2 expression *via* YTHDF2 mediated mRNA decay. 2.PJA2 suppressed the Wnt signaling pathway.	(8)
NUCB1	Suppressor	Down	1.NUCB1 decreased PC cell proliferation and GEM-induced autophagy and UPR. 2.NUCB1 increased PC cell apoptosis	OS↑	METTL3 YTHDF2	1.METTL3 KD decreased the m6A enrichment of NUCB1 5′UTR. 2.YTHDF2 recognized and bound to m6A-modified NUCB1 5′UTR and decreased NUCB1 mRNA stability and expression. 3.NUCB1 suppressed the antitumor role of GEM *via* inactivating ATF6.	(9)
FBXL5	Suppressor	Down	FBXL5 decreased PC cell migration, invasion, and iron level.	OS↑	ALKBH5	1.ALKBH5 reduced the m6A level of FBXL5 and its RNA stability. 2.ALKBH5 increased FBXL5 expression. 3.FBXL5 promoted the ubiquitination of IRP2 and SNAI1 proteins, forming a ALKBH5-FBXL5-IRP2/SNAI1 axis.	(65)
SLC25A37	Oncogene	Down	SLC25A37 increased THE mitochondrial iron level and dysregulation of immunometabolism.	OS↓	ALKBH5	1.ALKBH5 reduced the m6A level of SLC25A37 and regulated the alternative splicing of SLC25A37. 2.ALKBH5 increased SLC25A37 expression.	(65, 66)
SLC25A28	Oncogene	Down	SLC25A28 increased THE mitochondrial iron level and dysregulation of immunometabolism.	–	ALKBH5	1.ALKBH5 reduced the m6A level of SLC25A28 and its RNA stability. 2.ALKBH5 increased SLC25A28 expression.	(65, 66)

PC, pancreatic cancer; KD, knockdown; up, upregulation in PC, down, downregulation in PC; OS: overall survival; DFS: disease-free survival.

#### PER1

PERIOD1 (PER1) is a clock gene involved in circadian rhythm regulation, DNA damage, cell cycle, cell proliferation, and metastasis. The abnormal expression of PER1 has been shown in various types of cancers, and both oncogenic and tumor-suppressive roles of PER1 have been revealed (11, 60, 61, 67–76).

As reported, the expression of PER1 was significantly decreased in PC tissues as compared to the benign and adjacent normal tissues (11, 77) (**Table 1**). Moreover, the reduced expression of PER1 was associated with shorter OS of PC patients (11, 77). Analysis of RNA-seq and m6A-seq further revealed that PER1 was a downstream target of ALKBH5 (11) (**Table 1**). The expression of PER1 was increased in the presence of ALKBH5, whereas the deficiency of ALKBH5 led to a reduced PER1 expression, which was confirmed in PC through immunohistochemistry (IHC) and TCGA dataset analysis (11). The MeRIP-qPCR analysis revealed that the m6A level of PER1 was negatively regulated by the demethylase ALKBH5 (11) (**Table 1**; **Figure 2**). Additionally, YTHDF2 served as an m6A reader to trigger PER1 mRNA degradation. Furthermore, a positive feedback loop between ALKBH5 and PER1 was revealed, since PER1 attenuated the enhanced cell proliferation and invasion induced by ALKBH5 deficiency (11), while PER1 could in turn enhance p53 activation to elevate the ALKBH5 expression through the PER1–P53–ALKBH5 axis (11) (**Table 1**; **Figure 2**). Taken together, there is a novel positive feedback loop between ALKBH5 and PER1, and ALKBH5 triggers PER1 expression *via* in an m6A-YTHDF2-dependent manner to suppress PC progression. On the contrary, studies have also shown that PER1 was upregulated in PC tissues as compared to normal tissues (60). Furthermore, TNF-α treatment suppressed PER1 expression, and loss of PER1 suppressed cell proliferation and increased apoptosis of PC cells acting as an oncogene (60, 61). Therefore, PER1 might be a potential biomarker for PC prognosis and also a promising therapeutics target for PC treatment.

#### PERP

The P53 effector related to pmp-22 (PERP) was firstly reported as a transcriptional target of p53 involved in cell apoptosis (78). At present, a tumor-suppressive role of PERP has been confirmed in various cancers (62, 79–82).

Zhao et al. revealed that PERP was highly expressed in PC (83) (**Table 1**). PERP was further identified as a target of METTL14 through RNA sequencing and m6A sequencing. Acting as an m6A writer, METTL14 knockdown significantly decreased the m6A level of PERP-3′UTR, which stabilized the mRNA of PERP and further increased the expression of PERP. In contrast, METTL14 could reduce the stability and expression of PERP mRNA. Mechanistically, METTL14 deficiency promoted PERP expression in an m6A-YTHDF2-dependented manner, in which YTHDF2 mediated PERP mRNA degradation (**Table 1**; **Figure 2**). Moreover, PERP inhibition could rescue the decreased cell proliferation, migration, and invasion induced by METTL14 knockdown (7) (**Table 1**). The above findings suggest a tumor-suppressive role of PERP in the malignant progression of PC.

#### PIK3CB

p110β/PIK3CB is a p110 catalytic subunit of phosphoinositide 3-kinases (PI3Ks), which together with p110α/PIK3CA, p110δ/PIK3CB, and the p85 regulatory subunit formed class IA PI3Ks (84). Aberrant expression of PIK3CB has been found in multiple cancers and is involved in tumor cell growth, metabolism, angiogenesis, metastasis, and multidrug resistance (63, 64, 85–93).

As reported, PIK3CB expression was remarkably elevated in PC tissues and a high expression of PIK3CB predicted poor prognosis of PC patients (63, 64) (**Table 1**). PIK3CB promoted cell proliferation, migration, invasion, and tumorigenesis in PTEN-deficient PC cells both *in vitro* and *in vivo via* activating the Akt signaling pathway (64) (**Table 1**; **Figure 2**). Furthermore, m6A writers including METTL3, METTL14, and WTAP could positively regulate the m6A level of PIK3CB and then reduce its expression in both mRNA and protein levels (**Table 1**, **Figure 2**). On the contrary, FTO, as an m6A eraser, significantly reduced the m6A level of PIK3CB and subsequently enhanced the PIK3CB expression (**Table 1**; **Figure 2**). Moreover, YTHDF2 could interact with m6A-modified PIK3CB to decrease PIK3CB mRNA stability as well as its protein expression (64) (**Table 1**; **Figure 2**). In summary, m6A regulators (METTL3, METTL14, WTAP, FTO) inhibited PIK3CB expression *via* an m6A-YTHDF2-dependent way. The oncogenic effect of PIK3CB in PTEN-deficient PC, indicating that PIK3CB is an emerging therapeutic target for PC.

#### PJA2

Praja ring finger ubiquitin ligase 2 (PJA2) is a RING-H2-type E3 ubiquitin ligase, which was firstly identified as an axotomy-suppressed gene in nerve cells (94) and played key roles in regulating the cAMP-dependent activation of PKA (95). Emerging evidence has shown that PJA2 was aberrantly expressed across cancers and acts as an oncogene or a tumor suppressor in thyroid cancer (96), glioblastoma (97), and gastric cancer (98, 99).

As shown, PJA2 was significantly upregulated in PC cells (8). Furthermore, PJA2 underwent m6A regulation of FTO, since FTO increased while loss of FTO decreased the expression and m6A level of PJA2 (8) (**Table 1**). Subsequently, Zeng et al. demonstrated that YTHDF2 but not YTHDF1 acted as an m6A reader to mediate PJA2 mRNA degradation and its downregulation (**Table 1**; **Figure 2**). Moreover, PJA2 deficiency could rescue the FTO-induced suppression of cell growth and metastasis *via* inhibiting the Wnt signaling pathway (8) (**Table 1**). Taken together, PJA2 acted as a tumor suppressor to regulate PC malignant behaviors *via* the FTO–PJA2–Wnt axis in an m6A-YTHDF2-dependent way, indicating that PJA2 is a new promising molecular target for PC therapeutic treatment.

#### NUCB1

Nucleobindins including NUCB1 and NUCB2 are DNA- and calcium-binding proteins (100, 101), involved in the development of various cancers (9, 102–107). Currently, an oncogenic role of NUCB1 has been found in colon cancer (105) and breast cancer (106).

As regards PC, NUCB1 was downregulated in both mRNA and protein levels of PC tissues, and an obviously poor prognosis was observed in PC patients with a lower expression of NUCB1 (9) (**Table 1**). In contrast to the effect of NUCB1 in colon cancer and breast cancer, NUCB1 suppressed the cell growth and promoted the apoptosis of PC cells both *in vitro* and *in vivo*, while loss of NUCB1 in turn promoted PC cell growth and inhibited cell apoptosis (9) (**Table 1**). Hua et al. further clarified that METTL3 induced the m6A modification of NUCB1 5′UTR, in which YTHDF2 mediated m6A-modified NUCB1 mRNA degradation (9) (**Table 1**; **Figure 2**). Functionally, NUCB1 inhibited the cell proliferation and promoted the antitumor effect of gemcitabine (GEM) on PC cells *in vitro* and *in vivo (* 9). Moreover, NUCB1 also decreased autophagy and unfolded protein response (UPR)-induced by GEM *via* suppressing ATF6 activity (9) (**Table 1**; **Figure 2**). All the above findings demonstrate the m6A modulation of NUCB1 in an m6A–METTL3–YTHDF2-dependent way and a tumor-suppressive role of NUCB1 in PC progression.

#### FBXL5

F-box and leucine-rich repeat protein 5 (FBXL5) was firstly reported as a subunit of E3 ubiquitin ligase to promote the ubiquitination of p150^Glued^ (108). IRP2 and Snail1 have been identified as substrates of FBXL5 (109, 110). FBXL5 has been reported to contain a hemerythrin-like domain that binds to iron and oxygen, thereby being involved in iron homeostasis (109, 111). Likewise, iron and oxygen conditions could in turn regulate FBXL5 (109). Currently, both the oncogenic and tumor-suppressive roles of FBXL5 have been found across cancers including colon cancer and HCC (112–114).

Downregulation of FBXL5 has been detected in PC tissues, which was associated with poor prognosis of PC patients (65) (**Table 1**). FBXL5 was subsequently identified as a target of ALKBH5, which reduced the FBXL5 m6A level and enhanced mRNA stability to increase the FBXL5 expression (**Table 1**). Moreover, FBXL5 depletion successfully rescued the ALKBH5-mediated inhibition of intracellular iron accumulation, cell migration, and invasion through downregulating IRP2 and Snail (65). In a summary, FBXL5 served as a tumor suppressor in PC carcinogenesis through the ALKBH5–FBXL5–IRP2/SNAI1 axis in an m6A-dependent manner (**Figure 2**), indicating a potential role of FBXL5 in the diagnosis, prognosis, and target therapy of PC.

#### SLC25A28 and SLC25A37

Mitoferrin (MFRN) belongs to the mitochondrial solute carrier family (SLC25), located in the inner membrane (115). Mitoferrin consists of two isoforms: mitoferrin-1 (SLC25A37) and mitoferrin-2 (SLC25A28), which transport iron to the mitochondria (115). So far, both SLC25A37 and SLC25A28 have been involved in dysregulation of mitochondrial iron (116, 117). The tumor-suppressor roles of SLC25A37 and SLC25A28 have been confirmed since they were involved in tumor cell growth, ROS production, mitochondrial iron uptake, and ferroptosis (117–121).

SLC25A37 and SLC25A28 are lowly expressed in PC tissues (65). Li et al. found that a high expression of SLC25A37 but not SLC25A28 indicated shorter OS of PC patients (66) (**Table 1**). Huang et al. demonstrated that either SLC25A37 or SLC25A28 underwent m6A modification modulated by ALKBH5. Detailly, ALKBH5 demethylated SLC25A37 mRNA to modulate its alternative splicing (65) (**Table 1**). As for SLC25A28, ALKBH5 removed the m6A modification of SLC25A28 and thus promoted its mRNA stability and expression (65) (**Table 1**; **Figure 2**). More importantly, SLC25A37 or SLC25A28 was elevated by depletion of PINK1 or PARK2, enhanced mitochondrial iron level, and dysregulation of immunometabolism *via* the PINK1/PARK2–SLC25A37/SLC25A28–HIF1A–AIM2–HNGB1–CD274 axis and thereby triggered PC carcinogenesis (66). Therefore, SLC25A37 and SLC25A28 were regulated through both m6A-dependent and m6A-independent regulations in the modulation of PC progression.

### m6A Modification of Non-Coding RNAs in PC

#### WTAPP1

LncRNA WTAPP1, short for Wilms tumor 1-associated protein pseudogene 1, has been shown to play key roles in tumor cell proliferation, migration, invasion, and angiogenesis (122–124).

Here in PC, WTAPP1 was markedly overexpressed in tumor tissues, which was correlated with poor prognosis of PC patients (52) (**Table 2**). An oncogenic role of WTAPP1 was found in PC, showing that WTAPP1 enhanced PC cell proliferation and invasion both *in vitro* and *in vivo* (**Table 2**). Deng et al. further revealed that METTL3 mediated the m6A modification of WTAPP1, in which CCHC-type zinc finger nucleic-acid binding protein (CNBP) served as an m6A reader and stabilized the RNA of WTAPP1 (52) (**Table 2**). Moreover, the pseudogene WTAPP1 could enhance the translation of WTAP and activate the Wnt signaling, contributing to the malignant progression of PDAC (52) (**Table 2**, **Figure 2**). In summary, WTAPP1 may be a potential diagnostic and prognostic biomarker as well as a promising therapeutic target for PC.

**Table 2:** m6A modification of ncRNAs in pancreatic cancer.

Name	Role	Expression	Function	Clinical significance	m6A regulator	Mechanisms	Refs
WTAPP1	Oncogene	Up	WTAPP1 increased the PC cell growth and migration.	OS↓	METTL3 CNBP	1.METTL3 increased the m6A level and RNA expression of WTAPP1. 2.CNBP enhanced WTAPP1 RNA stability and subsequently accelerated WTAP translation and Wnt pathway activation.	(52)
LINC00857	Oncogene	Up	1.LINC00857 increased the PC cell proliferation, migration, and invasion. 2.LINC00857 reduced the PC cell apoptosis.	OS↓ DFS↓	METTL3	1.METTL3 increased the m6A modification, RNA stability, and expression of LINC00857 2.ceRNA: LINC00857/miR-150-5p/E2F3 axis. 3.ceRNA: LINC00857/miR-340-5p/TGFA axis 4.LINC00857 enhanced MET and then promoted STAT3 and CREB expression.	(125–127)
DANCR	Oncogene	Up	1.DANCR increased the PC cell proliferation, migration, invasion, stemness-like properties, and tumorigenesis. 2.DANCR inhibited PC cell apoptosis.	OS↓ PFS↓	IGF2BP2	1.IGF2BP2 recognized the m6A modification of DANCR and increased the DANCR RNA stability and expression 2.DANCR/miR-33b/MMP16 axis. DANCR sponge miR-33b to promote mmp16 expression 3.DANCR/miR-214-5p/E2F2 axis DANCR sponging miR-214-5p to promote E2F2 expression 4.DANCR/miR-135a/NLRP37 axis	(128–132)
KCNK15-AS1	Suppressor	Down	KCNK15-AS1 inhibited the PC cell proliferation, migration, and invasion.	DFS↑	ALKBH5	1.ALKBH5 decreased the m6a level of KCNK15-AS1 and enhanced the KCNK15-AS1 RNA stability and expression. 2.KCNK15-AS1inhibited KCNK15 translation. 3.KCNK15-AS1 enhanced PTEN to inhibit Akt signaling pathway.	(133, 134)
LIFR-AS1	Oncogene	Up	LIFR-AS1 KD suppressed the PC cell proliferation, migration, and invasion.	–	METTL3	1.METTL3 KD decreased the m6A level and RNA stability of LIFR-AS1 to reduce its expression. 2.LIFR-AS1 sponged miR-150-5p to promote VEGFA expression.	(135)
miR-25-3p	Oncogene	Up	miR-25-3p promoted the cell proliferation, migration, and invasion.	OS↓	METTL3 NKAP	1.CSC induced METTL3 expression *via* hypomethylation of METTL3. 2.METTL3 promoted miR-25-3p maturation *via* the NKAP-mediated m6A modification of pri-miR-25.	(53)
miR-30d	Suppressor	Down	miR-30d decreased the cell proliferation, migration, invasion, angiogenesis, and Warburg effect.	OS ↑ RFS↑ DFS↑	METTL3 METTL14 YTHDC1	1.METTL3/14 KD reduced the m6A enrichment of pri-miR-30d. 2.YTHDC1 induced the RNA decay of pri-miR-30d and increased the miR-30d expression. 3. miR-30d/RUNX1/GLUT1/HK1 axis. 4. miR-30d/SOX4/PI3K-Akt axis	(136, 137)

PC, pancreatic cancer; KD, knockdown; ncRNA, non-coding RNA; up, upregulation in PC, down, downregulation in PC; OS, overall survival; DFS, disease-free survival; PFS, progression-free survival; RFS, relapse-free survival.

#### LINC00857

Long non-coding RNA LINC00857 was firstly revealed to be highly expressed in lung cancer and indicated poor survival of lung cancer patients (138). Up to now, the upregulation of LINC00857 has been found in multiple types of cancers and is involved in tumor cell growth, migration, invasion, glycolysis, autophagy, and radiosensitivity (139–143).

As reported, LINC00857 was overexpressed in both PC cells and tissues, and its upregulation was associated with shorter OS and disease-free survival of PC patients (125–127) (**Table 2**). Studies have shown that LINC00857 could increase PC cell proliferation, migration, and invasion and decrease cell apoptosis (125–127) (**Table 2**). In regard to the molecular regulation of LINC00857, Meng et al. demonstrated that m6A writer METTL3 elevated both the m6A level and RNA stability of LINC00857 to promote LINC00857 expression, and then LINC00857 functioned as a ceRNA to regulate E2F3 expression through sponging miR-150-5p, contributing to the malignant development of PC cells (125) (**Table 2**; **Figure 2**). Moreover, Li et al. further demonstrated that LINC00857 could also sponge miR-340-5p to enhance the TGFA expression in PC cells and then accelerate PC cell migration and invasion (126) (**Table 2**, **Figure 2**). Additionally, LINC00857 could also upregulate MET, STAT3, and CREB expression to enhance PC cell proliferation (127). Above all, LINC00857 exerts an oncogenic role in PC and may provide a possible therapeutic target for PC treatment.

#### DANCR

Long non-coding RNA differentiation antagonizing non-protein-coding RNA (DANCR) was firstly identified as a progenitor differentiation suppressor in 2012 (144). Later, both the oncogenic and tumor-suppressive roles of DANCR have been identified across cancers (128, 145), participating in modulating tumor cell growth, stemness-like properties, EMT, and chemoresistance (145).

Consistent with previous reports in various cancers, DANCR was also highly expressed in both PC cells and tissues (129, 130) (**Table 2**). Moreover, a high expression of DANCR predicted poor clinical outcomes in PC (130) (**Table 2**). Luo et al. demonstrated that DANCR deficiency significantly decreased the cell proliferation, migration, and invasion of PC through the DANCR/miR-33b/MMP16 axis, in which DANCR served as a miR-33b sponge (129) (**Table 2**; **Figure 2**). Consistent with Yao et al.’s reports, Tang et al. also revealed that DANCR enhanced cell proliferation and invasion *via* the DANCR/miR-214-5p/E2F2 axis or DANCR/miR-135a/NLRP37 (130, 131) (**Table 2**; **Figure 2**). In addition to ceRNA regulation, DANCR also underwent m6A regulation. In detail, IGF2BP2 acted as an m6A reader and could recognize and bind to DANCR to enhance its RNA stability and expression, thereby facilitating PC cell proliferation and stemness-like properties (128) (**Table 2**; **Figure 2**). The above findings indicate the oncogenic role of DANCR and its potential clinical application in PC prognosis and treatment.

#### KCNK15-AS1

LncRNA KCNK15 and WISP2 antisense RNA 1 (KCNK15-AS1 or RP11-445H22.4) were firstly found to be upregulated in osteoarthritis (146). Subsequently, an abnormal expression of KCNK15-AS1 was also observed among cancers.

As reported, there was a remarkable downregulation of KCNK15-AS1 in both PC tissues and cell line, and patients with a low expression of KCNK-AS1 have shown shorter OS (133, 134) (**Table 2**). RNA m6A demethylase ALKBH5 could bind to KCNK15-AS1 and thus enhance its RNA stability and expression through eliminating m6A modification (133, 134) (**Table 2**; **Figure 2**). More importantly, KCNK15-AS1 suppressed KCNK15 translation through interacting with the 5′UTR of KCNK15, while KCNK15-AS1 also promoted PTEN expression and thus inhibited the Akt pathway, thereby suppressing PC cell proliferation, migration, and invasion (133, 134) (**Table 2**; **Figure 2**). In summary, KCNK15-AS1 regulated by ALKBH5-mediated m6A demethylation acted as a tumor suppressor to suppress the malignant progression of PC cells through the KCNK15–AS1/KCNK15 axis and KCNK15–AS1/PTEN/Akt axis, suggesting a promising therapeutic target for PC clinical treatment.

#### LncRNA LIFR-AS1

Leukemia inhibitory factor receptor antisense RNA 1 (LIFR-AS1) is a long non-coding RNA, which is transcribed from the LIFR gene. An abnormal expression of LIFR-AS1 has been found in various cancers and is involved in cancer development (147). The function of LIFR-AS1 is complex, since an antitumor role of LIFR-AS1 has been shown in glioma, breast cancer, and lung cancer, while an oncogenic role has been found in thyroid cancer, colorectal cancer, and osteosarcoma (147). Furthermore, both the oncogenic role and tumor suppressive role of LIFR-AS1 were revealed in gastric cancer (148, 149).

An obvious upregulation of LIFR-AS1 was observed in PC tissue and cell lines, which was correlated with poor clinical outcomes of PC patients (135) (**Table 2**). Knockdown of METTL3 reduced the m6A level of LIFR-AS1 and thus suppressed its RNA stability and expression (135) (**Table 2**, **Figure 2**). Moreover, a significant decrease in cell proliferation, migration, and invasion was observed following LIFR-AS1 inhibition (135) (**Table 2**). Additionally, a ceRNA regulation was also revealed showing that LIFR-AS1 could sponge miR-150-5p, thus activating downstream target VEGFA and promoting PC progression (135) (**Table 2**, **Figure 2**). These results revealed that LIFR-AS1 is an oncogenic gene in PC *via* the METTL3/LIFR-AS1/miR-150-5p/VEGFA axis in an m6A-dependent manner.

#### miR-25-3p

pri-miR-25 is the primary miRNA of miR-25-3p. miR-25 has been widely reported as an oncogenic miRNA. An abnormal expression of miR-25 has been found in multiple cancers (150, 151).

Here in PC, miR-25-3p was found to be highly expressed, which predicted the poor prognosis of PC patients (53) (**Table 2**). Zhang et al. further demonstrated that METTL3 induced by cigarette smoke condensate (CSC) could increase the m6A modification of pri-miR-25 *via* in a NKAP-dependent manner, in which NF-κB-associated protein (NKAP) functioned as an m6A reader of pri-miR-25 (53), thereby enhancing miR-25-3p maturation. Additionally, upregulated miR-25-3p could then activate the Akt signaling pathway through inhibiting the expression of its target-PHLPP2, thus promoting the cell migration and invasion of PC (53) (**Table 2**; **Figure 2**). In summary, the METTL3/miR-25-3p/PHLPP20/Akt axis exerts an oncogenic role in the carcinogenesis of PC patients who smoke.

#### miR-30d

miR-30d belongs to the miR-30 family, which is abnormally expressed across cancers. So far, both the oncogenic and antitumor roles of miR-30d have been revealed. miR-30d has been reported to remarkably suppress cell growth and metastasis of breast cancer (152), whereas upregulation of miR-30d enhanced tumor growth and angiogenesis of prostate cancer (153).

In PC, the expression of miR-30d was significantly decreased in both PC tissues and cell lines, which predicted a shorter OS, RFS, and DFS of PC patients (136, 137) (**Table 2**). More importantly, miR-30d overexpression inhibited PC cell growth, metastasis, and angiogenesis both *in vitro* and *in vivo (* 136, 137) (**Table 2**). miR-30d was further shown to be involved in glycolysis regulation since miR-30d decreased the lactic acid level, glucose uptake, and ATP level while miR-30d inhibition increased the lactic acid level, glucose uptake, and ATP level of PC (**Table 2**). miR-30d has m6A modification and is regulated by METTL3, METTL14, and YTHDC1. In detail, YTHDC1 significantly promotes the RNA degradation of pri-miR-30d and increases the expression of miR-30d, and knockdown of METTL3/14 significantly reduces the m6A enrichment of pri-miR-30d (136) (**Table 2**; **Figure 2**). Moreover, RUNX1 and SOX4 were identified as a downstream target of miR-30d. Hou et al. demonstrated that RUNX1 deficiency could attenuate miR-30d inhibition-induced PC cell growth, metastasis, angiogenesis, and glycolysis *via* the miR-30d/RUNX1/GLUT1/HK1 axis (136) (**Table 2**; **Figure 2**). Xu et al. further revealed that SOX4 overexpression successfully rescued the antitumor effect of miR-30d *via* the miR-30d/SOX4/PI3K-Akt axis (137) (**Table 2**; **Figure 2**). Taking all the above into consideration, miR-30d is shown to be modulated by YTHDC1-mediated m6A modification and there is a tumor-suppressive role of the miR-30d/RUNX1/GLUT1/HK1 axis and miR-30d/SOX4/PI3K-Akt axis in PC progression, providing a possible application of miR-30d as a diagnosis and prognosis biomarker and also a therapeutic target for PC treatment.

## Functions of m6A Regulators in Pancreatic Cancers

### Writers

#### WTAP

WTAP has been found to be highly expressed in PC tissue, which was correlated with shorter survival of PC patients (7, 52, 65, 154) (**Table 3**). As reported, WTAP triggered the cell proliferation, migration, and invasion and GEM resistance of PC cells, while knockdown of WTAP suppressed cell proliferation migration, and invasion and GEM resistance (52, 155) (**Table 3**).

**Table 3:** Functions of m6A writers in pancreatic cancer.

Name	Expression	Mechanism	Functions	Targets	Clinical significance	Refs
WTAP	Up	1.WTAP KD reduced the m6A level of PIK3CB and increased the PIK3CB expression *via* m6A-YTHDF2 mediated RNA decay of PIK3CB. 2.WTAP bound to and enhanced FAK mRNA stability to activate the FAK-PI3K-AKT and FAK-SRC-GRB2-ERK1/2 signaling pathway. 3.LncRNA WTAPP1 enhanced WTAP translation and then the WTAP-activated Wnt signaling pathway.	WTAP increased PC cell proliferation, migration, and invasion and GEM resistance.	**m6A-dependent:** PIK3CB. **m6A-indenpendent:** FAK.	OS ↓	(7, 52, 64, 65, 154, 155)
METTL3	Up	1.METTL3 KD reduced the m6A level of PIK3CB and increased PIK3CB expression *via* the m6A-YTHDF2-mediated RNA degradation of PIK3CB. 2.METTL3 KD reduced the m6A enrichment of NUCB1 5′UTR and increased the NUCB1 expression *via* YTHDF2-mediated RNA decay of NUCB1. 3.METTL3 KD reduced the m6A level of WTAPP1 and decreased its RNA stability and expression in an m6A-CNBP-dependent manner. 4.METTL3 KD reduced the m6A level, RNA stability, and expression of LIFR-AS1. 5.CSC induced the hypomethylation of the METTL3 promoter to enhance METTL3 expression, which increased the m6A level and expression of pri-miR-25 as well as miR-25-3p maturation in an NKAP-m6A-dependent way.	METTL3 promoted the cell proliferation, migration, invasion, stemness, and radio- and chemoresistance of PC cells.	**m6A-dependent**: PIK3CB, NUCB1, WTAPP1, LINC00857, LIFR-AS1, pri-miR-25, pri-miR-30d.	OS↓	(7, 9, 10, 52, 53, 64, 135, 156)
METTL14	Up	1.METTL14 KD reduced the m6A level of PIK3CB and PERP to increase their expression *via* m6A-YTHDF2-mediated RNA degradation. 2.METTL14 activated caspase3/8, mTOR pathway, and CDA expression. 3.SRFR5 regulated the AS of METTL14.	1.METTL14 increased PC cell proliferation, migration, invasion, metastasis, and chemoresistance. 2.METTL14 inhibited PC cell apoptosis and autophagy.	**m6A-dependent**: PIK3CB, PERP, pri-miR-30d.	OS↓	(7, 64, 157–159)
KIAA1429	Up	–	KIAA1429 KD inhibited PC cell proliferation.	–	OS↓	(160–162)
RBM15	Up	–	RBM15 KD inhibited PC cell proliferation,	–	OS↓, DFI↓ PFI↓, DSS↓	(163)

KD, knockdown; up, upregulation in PC; AS, alternative splicing; OS: overall survival; PFS: progression-free survival; DFI, disease-free interval; PFI, progression-free interval; DSS, disease-specific survival; -, no associated research.

As an m6A methylase, WTAP increased the m6A level of its target PIK3CB and enhanced the PIK3CB expression *via* an m6A-YTHDF2-mediated RNA decay of PIK3CB (64) (**Table 3**; **Figure 2**). Apart from m6A regulation, WTAP could also stabilize FAK mRNA and increase its expression through an m6A-indenpendent way, thereby activating the FAK-PI3K-AKT and FAK-SRC-GRB2-ERK1/2 signaling pathways (155) (**Table 3**; **Figure 2**). Moreover, GSK2256098, a specific FAK inhibitor, could attenuate WTAP-induced cell migration and invasion and GEM resistance of PC cells (155). As a key modulator to affect its downstream target genes, WTAP could also be regulated by its upstream genes. Deng et al. have shown that LncRNA WTAPP1 could recruit EIF3B to enhance WTAP translation, and then WTAP activated the Wnt signaling pathway, forming a functional WTAPP1/WTAP/Wnt axis[75] (**Table 3**; **Figure 2**). Additionally, knockdown of WTAP attenuated WTAPP1-induced PC cell proliferation, migration, and invasion (52) (**Table 3**). All the above findings indicate the oncogenic role of WTAP in PC in an m6A-dependent and m6A-independent way as well as the potential application of WTAP in PC prognosis and targeted therapy.

#### METTL3

Similar to WTAP, METTL3 was also shown to be upregulated in PC, which was correlated with shorter OS of PC patients (7, 10) (**Table 3**). According to the studies, METTL3 deficiency decreased PC cell proliferation, migration, invasion, stemness, and radio- and chemoresistance (10, 156) (**Table 3**). Acting as an m6A methylase, METTL3 knockdown obviously reduced the total RNA m6A level of PC cells (10). Several m6A-regulated targets of METTL3 have been identified, such as PIK3CB, NUCB1, WTAPP1, LINC00857, LIFR-AS1, pir-miR-25, and pri-miR-30d (**Table 3**).

Firstly, METTL3 inhibition reduced the m6A level of PIK3CB and increased the PIK3CB expression *via* YTHDF2-mediated mRNA decay (64). Later, Hua et al. found that the m6A enrichment of the NUCB1 5′UTR was notably decreased upon knockdown of METTL3 *via* in an m6A-YTHDF2-dependent way (9) (**Table 3**; **Figure 2**). In addition to coding RNAs, METTL3 also mediated the m6A regulation of non-coding RNAs. For instance, loss of METTL3 significantly reduced the m6A level of WTAPP1 as well as its expression, in which m6A reader CNBP enhanced the mRNA stability of WTAPP1 (52) (**Table 3**; **Figure 2**). Furthermore, METTL3 deficiency also decreased the m6A level and RNA stability of LINC00857 and LIFR-AS1, resulting in their downregulation (135) (**Table 3**; **Figure 2**). Smoke was a high-risk factor of PC, and smokers were reported to have a two-fold higher risk of PC than non-smokers (164). Interestingly, there was a significant upregulation of METTL3 in smokers as compared with non-smokers (53). Zhang et al. have further shown that cigarette smoke condensate (CSC) promoted METTL3 expression through hypomethylating the promoter of METTL3 (53), which enhanced the m6A level and expression of pri-miR-25 and miR-25-3p maturation *via* in an m6A-NKAP-dependent manner, in which NF-κB-associated protein (NKAP) functioned as an m6A reader of pri-miR-25 (53) (**Table 3**; **Figure 2**). Additionally, METTL3 was also shown to affect the m6A enrichment of pri-miR-30d (136). In summary, METTL3 served as an oncogene in PC progression and provides a possible prognosis biomarker and therapeutic target for PC.

#### METTL14

An obvious upregulation of METTL14 has been found in both RNA and protein levels in PC tissues (7, 157) (**Table 3**). Patients with a higher expression of METTL14 have shown shorter OS (7) (**Table 2**). As mentioned before, METTL14 was involved in various cellular processes of PC cells, since METTL14 remarkably promoted PC cell proliferation, migration, and invasion; metastasis; cisplatin resistance; and GEM resistance while it inhibited the apoptosis and autophagy of PC cells (7, 157–159) (**Table 3**).

It was shown that loss of METTL14 increased cisplatin-induced cell apoptosis and autophagy by activating caspase3/8 and mTOR pathway, thereby enhancing the antitumor effect of cisplatin (157) (**Table 3**; **Figure 2**). Interestingly, GEM treatment specifically upregulated the expression of METTL14, without changes in other m6A regulators (158). However, GEM-induced METTL14 could in turn increase the GEM resistance *via* promoting CDA expression both *in vitro* and *in vivo (* 158) (**Table 3**; **Figure 2**). Additionally, SRFR5 was shown to regulate the alternative splicing of METTL14, which formed a SRSF5–METTL14 axis to enhance PC cell growth and metastasis *in vitro* and *in vivo (* 159) (**Table 3**). For m6A regulation, Tian et al. revealed that loss of METTL14 suppressed the m6A level and promoted the expression of PIK3CB *via* m6A-YTHDF2-mediated RNA decay of PIK3CB (64) (**Table 3**; **Figure 2**). Moreover, Wang et al. demonstrated that METTL14 deficiency also decreased the m6A level and thus increased the expression of PERP through m6A-YTHDF2-mediated degradation of PERP mRNA (7), resulting in cell growth and metastasis of PC (**Table 3**; **Figure 2**). In addition, METTL14 knockdown also deceased the m6A enrichment of pri-miR-30d (136). The above results suggest that both the m6A-dependent and m6A-independent regulation of METTL14 are involved in the carcinogenesis of PC, and METTL14 is a promising diagnosis and prognosis biomarker and chemotherapy resistance target for PC treatment.

#### KIAA1429

Vir-like m6A methyltransferase-associated (VIRMA, also named KIAA1429) was significantly upregulated in PC tissues as compared to normal tissues (160) (**Table 3**). Moreover, KIAA1429 was revealed as an independent risk factor for PC prognosis (165), and high expression of KIAA1429 was associated with shorter OS of PC patients (161, 162) (**Table 3**). Depletion of KIAA1429 remarkably reduced the cell proliferation of PC cells (162), indicating an oncogenic role of KIAA1429 in PC (**Table 3**; **Figure 2**).

#### RBM15

RNA-binding motif protein 15 (RBM15) has been identified as a methylase during m6A modification. According to TCGA and GTEx databases, RBM15 was highly upregulated in PC tissues and loss of RBM15 suppressed the cell proliferation of PC cells (163) (**Table 3**). Moreover, PC patients with a high expression of RBM15 have shown decreased OS, DFI, PFI, and DSS (163) (**Table 3**). Additionally, a highly correlated relationship between RBM15 expression and immune checkpoint markers was also revealed. The above findings suggest a favorable application of RBM15 in the prognosis and immunotherapy of PC.

### Readers

#### IGF2BP1

It has been shown that an upregulation of IGF2BP1 was observed in PC tissues (166), which was associated with shorter OS of PC patients (166) (**Table 4**). Knockdown of IGF2BP1 dramatically reduced cell proliferation and induced G1 cell-cycle arrest and apoptosis (166, 167) (**Table 4**). IGF2BP1, an RNA-binding protein, attenuated the Linc00261-induced suppression of c-myc RNA stability through binding to Linc00261 (168) (**Table 4**; **Figure 2**). Moreover, IGF2BP1 cooperated with LncNEAT1 to increase the RNA stability of ELF3 and enhanced PC cell proliferation, migration, and invasion (167) (**Table 4**; **Figure 2**). In addition, miR-494 could target IGF2BP1 to suppress IGF2BP1 expression and then IGF2BP1 promoted PC progression *via* activating the Akt pathway (166) (**Table 4**; **Figure 2**). Therefore, IGF2BP1 might serve as a potential therapeutic target and prognostic biomarker for PC.

**Table 4:** Functions of m6A readers and erasers in pancreatic cancer.

Name	Expression	Mechanism	Functions	Targets	Clinical significance	Refs
IGF2BP1	Up	1.IGF2BP1 increased the RNA stability of c-myc and ELF3. 2.miR-194 targeted IGF2BP1 to inhibit IGF2BP1 expression.	IGF2BP1 KD inhibited PC cell proliferation and induced G1 cell cycle arrest and apoptosis.	**m6A-independent**: c-myc, ELF3	OS↓	(166–168)
IGF2BP2	Up	1.IGF2BP2 promoted GLUT1 expression *via* stabilizing GLUT1 mRNA. 2. miR-141 downregulated IGF2BP2 to activate the PI3K-Akt pathway. 3. IGF2BP2 enhanced DANCR expression *via* binding to and stabilizing m6A-modified DANCR	IGF2BP2 promoted PC cell growth, invasion, aerobic glycolysis, and stemness-like properties.	**m6A-dependent**: DANCR. **m6A-independent**: GLUT1.	OS↓	(128, 160–162, 169–173)
IGF2BP3	Up	1.Lin28B/Let7 targets IGF2BP3 to downregulate IGF2BP3.	IGF2BP3 enhanced the PC cell proliferation, migration, invasion, metastasis, and stemness-like properties.	–	OS↓ PFS↓	(162, 172, 174–180)
YTHDF1	Up	–	–	–	–	(181, 182)
YTHDF2	Up	1.YTHDF2 acted as an m6A reader and induced the RNA degradation of m6A-modified PER1, PERP, PIK3CB, PJA2, and NUCB1. 2.YTHDF2 KD inhibited the Akt/GSK3β/CyclinD1 signaling pathway and activated the YAP signaling pathway.	YTHDF2 KD inhibited cell growth and promoted cell migration, invasion, and EMT.	**m6A-dependent**: PER1, PERP, PIK3CB, PJA2, NUCB1	OS↓ Advanced stage	(7–9, 11, 64, 65, 181–183)
YTHDF3	Up	–	–	–	–	(65)
YTHDC1	Down	YTHDC1 promoted miR-30d maturation through enhancing the m6A-dependent RNA degradation of pri-miR-30d.	YTHDC1 inhibited PC cell growth.	**m6A-dependent**: pri-miR-30d	OS↑ DFS↑	(136)
YTHDC2	Down	–	–	–	–	(136)
HNRNPC	–	–	HNRNPC KD inhibited PC cell proliferation.	–	OS↓	(162)
CNBP	–	CNBP enhanced RNA stability of WTAPP1.	–	–	–	(52)
ALKBH5	Down	1.ALKBH5 reduced the m6A level of PER1 to enhance the PER1 expression *via* in an m6A-YTHDF2-dependent way, forming a positive feedback loop of the ALKBH5/PER1/p53/ALKBH5 axis. 2.ALKBH5 reduced the m6A level while increasing the expression of WIF-1 and KCNK-AS1. 3.ALKBH5 promoted FBXL5 and SLC25A28 expression and also modulated the alternative splicing of SLC25A37 *via* m6A modification.	1.ALKBH5 decreased cell proliferation, migration, invasion, and GEM-resistance. 2.ALKBH5 modulated PC cell iron metabolism.	**m6A-dependent:** WIF-1, PER1, FBXL5, SLC25A28, SLC23A37, KCNK15-AS1.	OS↑ OS↓	(11, 56, 65, 133, 134, 184)
FTO	Down	1.FTO reduced the global m6A level of PC cells. 2.FTO reduced the m6A level of PJA2 and promoted the PJA2 expression *via* m6A-YTHDF2-mediated RNA degradation, suppressing the Wnt pathway.	FTO inhibited cell proliferation, migration, and invasion.	**m6A-dependent**: PJA2	OS↑ Lymph node metastasis↓	(8, 65)
	Up	FTO promoted c-myc expression *via* enhancing c-myc mRNA stability.	FTO KD reduced PC cell proliferation and enhanced apoptosis.	**m6A-independent**: c-myc	–	(185)

KD, knockdown; up, upregulation in PC, down, downregulation in PC; AS, alternative splicing; OS, overall survival; DFS, disease-free survival; PFS, progression-free survival; -, no associated research.

#### IGF2BP2

Consistent with IGF2BP1, IGF2BP2 was also significantly upregulated in PC tissues and cell lines (128, 169–172) (**Table 4**). A high expression of IGF2BP2 predicted a shorter OS of PC patients (128, 160–162, 169–173) (**Table 4**). Knockdown of IGF2BP2 significantly reduced PC cell growth and invasion (128, 162, 169, 171, 172) (**Table 4**). It has been reported that IGF2BP2 promotes the aerobic glycolysis of PC cells by binding to and stabilizing GLUT1 mRNA (169) (**Table 4**; **Figure 2**). IGF2BP2 was subsequently revealed as a potential target of miR-141 and to be involved in miR-141-induced PC cell growth *via* activating the PI3K-Akt signaling pathway (171) (**Table 4**; **Figure 2**). Acting as an m6A reader, IGF2BP2 could bind to DANCR to increase RNA stability *via* in an m6A-dependent manner and enhance the cell proliferation and stemness-like properties of PC cells (128) (**Table 4**; **Figure 2**). Thus, IGF2BP2 plays an oncogenic role in PC.

#### IGF2BP3

A high expression of IGF2BP3 in both PC tissues and cell lines was observed (172, 174, 175) (**Table 4**) and was correlated with shorter OS as well as PFS of PC patients (162, 172, 174–177) (**Table 4**). Knockdown of IGF2BP3 reduced the cell proliferation, migration, and invasion of PC cells (172, 178, 179). Mechanism-wise, IGF2BP3, located in cytoplasmic stress granules along with its downstream targets ARF6 and ARHGE4, promoted cell protrusion formation and enhanced PC cell invasion and metastasis (179, 180). A genome-wide analysis upon IGF2BP3 knockdown has further shown that IGF2BP3 was strongly correlated with genes regulating cell migration, proliferation, and adhesion (178). Moreover, IGF2BP3 was identified as a target of Lin28B/Let7 and enhanced the cell growth and stemness-like properties of PC cells (177) (**Table 4**; **Figure 2**). Overall, IGF2BP3 might be a promising diagnostic and prognostic biomarker as well as therapeutic target for PC.

#### YTHDFs

YTHDF1, YTHDF2, and YTHDF3 were upregulated in PC tissues (65, 181–183) (**Table 4**). Among these YTHDF family genes, YTHDF2 has been extensively studied in PC, while few studies have reported the roles of YTHDF1 and YTHDF3 in PC. As previously reported, PC patients with a higher expression of YTHDF2 have shown a shorter OS (181) and advanced stage (183) (**Table 4**). Chen et al. found that knockdown of YTHDF2 inhibited cell growth through inhibiting the Akt/GSK3β/CyclinD1 signaling pathway (183) (**Table 4**; **Figure 2**). However, an enhancement of cell migration, invasion, and EMT was also observed upon YTHDF2 deficiency (183). Furthermore, loss of YTHDF2-mediated YAP signaling activation may participate in PC cell EMT (183) (**Table 4**; **Figure 2**). Referring to m6A regulation, YTHDF2 served as an m6A reader which could recognize and bind to m6A-modified PIK3CB, PERP, PER1, PJA2, and NUCB1 RNA, thereby mediating their RNA degradation (7–9, 11, 64) (**Table 4**; **Figure 2**). The above findings indicate the critical roles of YTHDF2 in PC malignant progression.

#### YTHDCs

YTHDC1 and YTHDC2 were downregulated in PC tissues when compared to normal tissues (136) (**Table 4**). The upregulation of YTHDC1 predicted the longer OS and relapse-free survival (RFS) of PC patients (136) (**Table 4**). As an m6A reader, YTHDC1 triggered the degradation of pri-miR-30d and enhanced the maturation of miR-30d in an m6A-dependent manner (136) (**Table 4**; **Figure 2**). Finally, YTHDC1 was further found to suppress cell growth induced by miR-30d inhibition (136). Therefore, YTHDC1 might be a possible biomarker for PC prognosis and targeted therapy due to its antitumor effect.

#### HNRNPC

Few studies have reported the function of HNRNPC in PC. Hou et al. have shown that knockdown of HNRNPC significantly reduced the cell proliferation of PC cells (162) (**Table 4**). A high expression of HNRNPC was associated with a shorter OS of PC patients (162) (**Table 4**).

### Erasers

#### FTO

A contradictory expression of FTO has been reported in PC. On the one hand, Tang et al. found that FTO was highly expressed in both PC tissues and cell lines (185) (**Table 4**). Loss of FTO inhibited cell proliferation and also enhanced the apoptosis of PC cells (185). Meanwhile, a significantly elevated m6A level of PC cells was detected after FTO knockdown through m6A dot-blot (185). Furthermore, FTO could interact with c-myc and enhance the expression and mRNA stability of c-myc, forming a functional FTO/c-myc axis (185) (**Table 4**; **Figure 2**). On the other hand, downregulation of FTO in PC tissues was also observed (8, 65) (**Table 3**). Furthermore, a low expression of FTO predicted a shorter OS of PC patients (8). FTO suppressed PC cell proliferation, migration, and invasion (8). Acting as an m6A demethylase, FTO reduced while FTO deficiency enhanced the total RNA m6A level of PC cells (8). Moreover, FTO reduced the m6A level of PJA2 and increased PJA2 expression *via* YTHDF2-mdeidated RNA degradation of PJA2, thereby suppressing the Wnt pathway and forming a functional FTO/YTHDF2/PJA2/Wnt axis to inhibit PC malignant progression (8) (**Table 4**; **Figure 2**). Taken together, FTO exerted both oncogenic and antitumor roles in the carcinogenesis of PC.

#### ALKBH5

Unlike the m6A writer expression in PC, ALKBH5 expression is significantly reduced in PC tissues, and PC patients with a low expression of ALKBH5 have shown a shorter OS (11, 56, 65). In contrast, Cho et al. revealed that a high expression of ALKBH5 was associated with a shorter OS of PC patients (184) (**Table 4**). So far, ALKBH5 has been reported to negatively regulate PC cell proliferation, migration, and invasion; iron metabolism; and GEM resistance (11, 56, 65, 133, 134) (**Table 4**). Currently, several m6A targets of ALKBH5 have been identified, such as WIF-1, PER1, FBXL5, SLC25A28, SLC25A37, and KCNK15-AS1. As reported, ALKBH5 reduced the m6A level of PER1 and then increased PER1 expression *via* in an m6A-YTHDF2-dependent way (11) (**Table 4**; **Figure 2**). Moreover, PER1 could in turn increase the ALKBH5 expression through activating p53 (11), suggesting a positive feedback loop between ALKBH5 and PER1 in promoting tumor growth and metastasis of PC (**Table 4**; **Figure 2**). Tang et al. found that ALKBH5, downregulated by GEM treatment, could also decrease the m6A level of WIF-1 and promote its expression to suppress the Wnt pathway, leading to PC cell growth, metastasis, and GEM resistance (56) (**Table 4**; **Figure 2**). In addition, ALKBH5 modulated the RNA stability of FBXL5 and SLC25A28, as well as the alternative splicing of SLC25A37 in an m6A-dependent manner (65), and reduced the cell migration and invasion and the intracellular iron level, thus preventing PC progression (65) (**Table 4**; **Figure 2**). He et al. further demonstrated that ALKBH5 remarkably enhanced the KCNK15-AS1 expression through decreasing the m6A level and stabilizing the KCNK15-AS1 mRNA, thereby suppressing cancer development (133, 134) (**Table 4**; **Figure 2**). Therefore, the above results revealed a tumor-suppressive role of ALKBH5 in PC, indicating a possible application of ALKBH5 for PC prognosis and chemoresistance prediction.

## Conclusions and Perspectives

In recent years, the molecular mechanisms of genetic and epigenetic regulation have been extensively studied in the occurrence and progression of PC. Notably, increasing attention has been paid to the m6A modifications in PC development. Here in this review, we focused on the function and molecular mechanism of m6A regulators and m6A-regulated genes. We summarized that m6A modifications exerted their functions mainly in two ways. Firstly, m6A modifications modulate mRNA methylation to affect their RNA stability as well as protein expression (**Table 1**; **Figure 2**). Secondly, m6A modifications regulate the methylation of ncRNAs including long non-coding RNAs (LncRNAs) and miRNAs and alter the ncRNA expression to participate in PC carcinogenesis (**Table 2**; **Figure 2**). In spite of the above findings, the molecular mechanism of m6A regulation in PC remains largely unknown. Increasing comprehensive and in-depth studies are required to elucidate the critical roles of m6A modification in the malignant progression of PC and to further identify novel promising diagnostic and prognostic biomarkers as well as therapeutic targets for PC, and finally explore their possible clinical applications. Moreover, further research is also required to be done to illustrate the m6A modulation in higher-risk factors of PC, such as smoking, obesity, diabetes, and chronic pancreatitis. Therefore, systematic and comprehensive studies are urgently needed to clarify the interplay between m6A regulation and PC malignant progression, paving the way for exploring more approaches for PC treatment.

## Author Contributions

XH, WSu, QX, and KT contributed to the idea and the manuscript. XL, WF, WSun, and QL collected the literature. JG contributed to the figure preparation. All authors contributed to the article and approved the submitted version.

## Funding

This study was supported by the National Natural Science Foundation of China (82103295, 81801642), Natural Science Foundation of Zhejiang Province (LQ22H160062), and Medical and Health Science Technology Project of Zhejiang Province (2019RC105, 2022KY516).

## Conflict of Interest

The authors declare that the research was conducted in the absence of any commercial or financial relationships that could be construed as a potential conflict of interest.

## Publisher’s Note

All claims expressed in this article are solely those of the authors and do not necessarily represent those of their affiliated organizations, or those of the publisher, the editors and the reviewers. Any product that may be evaluated in this article, or claim that may be made by its manufacturer, is not guaranteed or endorsed by the publisher.
